# Fully Automated 3D Cardiac MRI Localisation and Segmentation Using Deep Neural Networks

**DOI:** 10.3390/jimaging6070065

**Published:** 2020-07-06

**Authors:** Sulaiman Vesal, Andreas Maier, Nishant Ravikumar

**Affiliations:** 1 Pattern Recognition Lab, Computer Science, Friedrich-Alexander-Universität Erlangen-Nürnberg, 91052 Erlangen, Germany; andreas.maier@fau.de (A.M.); n.ravikumar@leeds.ac.uk (N.R.); 2 Centre for Computational Imaging and Simulation Technologies in Biomedicine (CISTIB), School of Computing, LICAMM Leeds Institute of Cardiovascular and Metabolic Medicine, School of Medicine, University of Leeds, Leeds LS2 9JT, UK

**Keywords:** cardiac MRI, deep neural network, CNN, multistage segmentation, MRI segmentation, cardiovascular diseases

## Abstract

Cardiac magnetic resonance (CMR) imaging is used widely for morphological assessment and diagnosis of various cardiovascular diseases. Deep learning approaches based on 3D fully convolutional networks (FCNs), have improved state-of-the-art segmentation performance in CMR images. However, previous methods have employed several pre-processing steps and have focused primarily on segmenting low-resolutions images. A crucial step in any automatic segmentation approach is to first localize the cardiac structure of interest within the MRI volume, to reduce false positives and computational complexity. In this paper, we propose two strategies for localizing and segmenting the heart ventricles and myocardium, termed multi-stage and end-to-end, using a 3D convolutional neural network. Our method consists of an encoder–decoder network that is first trained to predict a coarse localized density map of the target structure at a low resolution. Subsequently, a second similar network employs this coarse density map to crop the image at a higher resolution, and consequently, segment the target structure. For the latter, the same two-stage architecture is trained end-to-end. The 3D U-Net with some architectural changes (referred to as 3D DR-UNet) was used as the base architecture in this framework for both the multi-stage and end-to-end strategies. Moreover, we investigate whether the incorporation of coarse features improves the segmentation. We evaluate the two proposed segmentation strategies on two cardiac MRI datasets, namely, the Automatic Cardiac Segmentation Challenge (ACDC) STACOM 2017, and Left Atrium Segmentation Challenge (LASC) STACOM 2018. Extensive experiments and comparisons with other state-of-the-art methods indicate that the proposed multi-stage framework consistently outperforms the rest in terms of several segmentation metrics. The experimental results highlight the robustness of the proposed approach, and its ability to generate accurate high-resolution segmentations, despite the presence of varying degrees of pathology-induced changes to cardiac morphology and image appearance, low contrast, and noise in the CMR volumes.

## 1. Introduction

Cardiovascular diseases (CVDs) and other cardiac pathologies are the leading cause of death in Europe and the USA [1,2]. Timely diagnosis and post-treatment follow-ups are imperative for improving survival rates and delivering high-quality patient care. These steps rely heavily on numerous cardiac imaging modalities, which include CT (computerized tomography), coronary angiography, and cardiac MRI. Cardiac MRI is a non-invasive imaging modality used to detect and monitor cardiovascular diseases. Consequently, quantitative assessment and analysis of cardiac images is vital for diagnosis and devising suitable treatments. The reliability of quantitative metrics that characterize cardiac functions such as myocardial deformation and ventricular ejection fraction, depends heavily on the precision of the heart chamber segmentation [3]. Manual segmentation is an error-prone, time-consuming, and tedious task, which requires roughly 20 min per ventricle for an expert, even with assistance from a suitable software [4,5]. Additionally, manual approaches suffer from high inter- and intra-rater variability, affecting the reproducibility, interpretability, and reliability in the diagnostic workflow. Consequently, the development of an accurate, efficient, and automatic approach for cardiac segmentation is highly desirable.

Among the four heart chambers, imaging of the left ventricle (LV), right ventricle (RV) and left atrium (LA) are of great importance and interest, as several cardiac diseases affect the morphology and function of these structures, such as atrial fibrillation (AF), cardiomyopathy, etc. [6]. An essential step in any automatic segmentation approach is to first localize the cardiac structure of interest within the MRI volume, to reduce false positives and computational complexity, by limiting the computation domain to the vicinity of the target structure.

Robust and accurate anatomy localization is an active area of research, and one of significant interest, due to the ever-increasing numbers of 3D medical images acquired in clinics worldwide. Such approaches enable faster data navigation and visualization of target structures, saving valuable time for the radiologist [7]. Additionally, they help initialize subsequent segmentation or registration algorithms, thereby significantly improving their performance, as demonstrated in the previous studies [8]. Overall, it is a crucial component in the design of an accurate computer-aided-diagnosis (CAD), and clinical decision support workflow.

## 2. Related Work

Traditional approaches to cardiac MRI segmentation were model-based, or hybrid techniques, which employed deformable [9], active contour [10], or statistical shape models [11], in combination with thresholding and morphological operations. A variety of atlas-based label fusion approaches [12,13,14] have also been proposed for the same task.

Recently, there have been tremendous improvements in cardiac MRI segmentation [15], and in medical image segmentation in general, using deep convolutional network architectures [16,17]. These networks are usually encoder–decoder type architectures, where the role of the decoder network is to project the encoded low-resolution feature maps, to high-resolution ones for pixel-wise classification. Encoder–decoder-based CNN architectures have been used extensively for several medical image segmentation applications. For example, Zotti et al. [15] proposed an extension to the U-Net. A cardiac shape priorly employed to accurately localize the endo- and epicardium of the left ventricle and the endocardium of the right ventricle. It achieved using a multi-resolution grid architecture that learned both low and high-level features to register the shape prior to a short-axis cardiac MRI.

Xiong et al. [18] proposed AtriaNet for segmenting left atria in 3D late gadolinium-enhanced (LGE)-MRI. Their method consists of a multi-scale CNN architecture, which captures both local and global atrial morphology. AtriaNet achieved a dice score of 0.940 and 0.942 for the LA epicardium and endocardium. A spectrum-based method developed by Zhong et al. to locate the left ventricle using a discrete Fourier Transform [19]. Harmonic images of all frequencies were analyzed visually and quantitatively to determine different spectral patterns for the left and right ventricles. The first and fifth harmonic images were selected to perform an anisotropically-weighted circular Hough detection.

Omega-net proposed by Vigneault et al. [20] for the ACDC challenge [21], comprises multiple steps—any given input image is first segmented using the network; subsequently, the features learned during this initial step are used to predict the parameters needed to transform the input image into a canonical orientation; and finally, the image is segmented once again in the transformed canonical space. A two-stage CNN-based approach proposed by Yang et al. [22] for left atrium segmentation, which comprised a detection module, and a subsequent segmentation step using transfer learning, a novel focal loss, and a recursive training strategy. Similar approaches used in CT [23,24]. Furthermore, Isensee et al. [25] created an ensemble of 2D and 3D U-Net architectures (with residual connections along with the up-sampling layers) for the heart segmentation and disease diagnosis challenges hosted as part of the STACOM workshop at MICCAI 2017. Here, due to the large slice thickness of the input images, pooling and upsampling operations were performed only in the short axis plane, for the 3D network. Moreover, due to memory constraints, the 3D network was designed using a smaller number of feature maps.

In a more clinically adapted approach [26] employed a deep learning model for cardiac motion analysis and human survival prediction. They used MR sequences to train a multi-task convolutional neural network for image segmentation and anatomical keypoint extraction. The key points were employed for further motion estimation. Moreover, based on the segmentation output, they extracted latent representation to predict the survival rate of the patient. Ruijsink et al. [27] proposed a deep-learning framework based on biventricular segmentation (3975 subjects) in long-axis and short-axis views and a post-analysis quality control to detect erroneous results. They cropped images automatically based on the center of the MR images to find the region of interest only. Moreover, Fahmy et al. [28] proposed a method for automatically quantifying LV mass and scar volume on LGE in patients with cardiomyopathy. However, most of these methods are based on 2D models.

To address the challenges as yet unmet by existing methods such as model complexity, robustness to noise, and lack of incorporating temporal information, we propose a fully 3D automatic deep learning model for localizing and segmenting ventricular anatomies in cardiac MRI. The objective is to facilitate the generation of accurate, high-resolution segmentation, by detecting the cardiac anatomy first. To this end, a multi-stage fully convolutional neural network was designed, wherein the first stage estimates a coarse density map localizing the structure of interest. It acts as an attention map to guide the second network (segmentation) to focus on the region of interest (ROI). Two strategies to focus the second stage of the network using the estimated attention map are investigated, namely, a ‘multi-stage’ and an ‘end-to-end’ approach. For the former, the attention map enables an ROI to be cropped from the input volume, maintaining the same image resolution. The cropped ROI is subsequently used by the second stage of the network to segment the structure(s) of interest at the original resolution of the image. As the training of each step is disjoint, we call it as ‘multi-stage’. With the ‘end-to-end’ approach, the attention map estimated by the first stage of the network is added to a downsampled (low-resolution) version of the original image and used as input for the second stage of the network.

We applied our framework to the challenging task of cardiac MRI segmentation to highlight the efficacy of the proposed contributions. It is considered a challenging segmentation task due to low and varied contrast at tissue-boundaries, and large variability in the morphology of cardiac structures across healthy subjects, and patients suffering from different cardiac diseases. We evaluated our framework on two publicly available datasets: Automatic Cardiac Segmentation Challenge (ACDC) STACOM 2017 [21]; and Left Atrium Segmentation Challenge (LASC) STACOM 2018 (http://atriaseg2018.cardiacatlas.org/) datasets.

## 3. Methods

In this section, first we describe our proposed methods for cardiac MRI localisation and segmentation in detail, and then, explain the performed experiments on different datasets.

**Figure 1:**
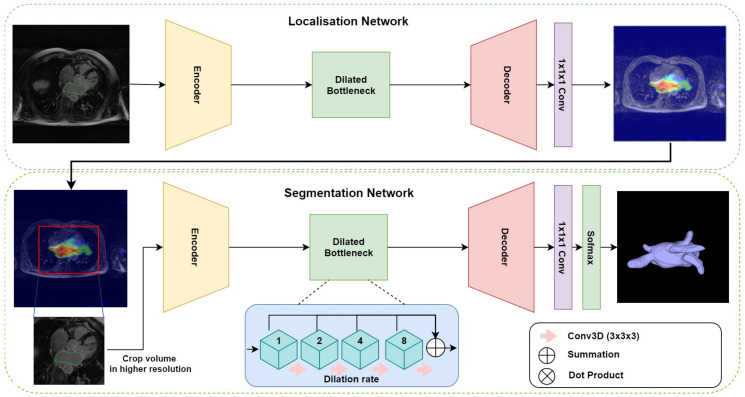
Overall pipeline for the proposed multi-stage network architecture, which depicts an example late gadolinium-enhanced (LGE)-MRI scan with its corresponding intermediate GCAM result. Both encoder–decoder networks used for localisation and segmentation have similar architectures, except in terms of complexity (i.e., number of convolution kernels used in each encoder–decoder block).

The proposed multi-stage network consists of three modules: an encoder–decoder for localisation, gradient-weighted class activation maps (GCAM) for density map generation, and an encoder–decoder for final segmentation. The overall architecture is depicted in Figure 1. The first network takes the downsampled MRI volumes as input and produces coarse feature maps for each volume. The GCAM module estimates the gradient-weighted activation maps of the object of interest from these feature maps and subsequently, a fixed-size sub-volume is cropped based on the GCAM density map. The outputs of this module are then used by the second encoder–decoder network, to generate the final segmentation. An end-to-end variant of this network was also designed, wherein, a dot product of the coarse feature map estimated from the first module and the input volume is computed to generate an ‘attention’ map. This attention map is used in place of the GCAM module and cropping function employed by the multi-stage network and forms the input for the subsequent segmentation network.

For both the localization and segmentation networks, we developed a 3D dilated residual U-Net (3D DR-UNet) as the backbone architecture. It is motivated by the success of the U-Net [17] which uses an encoder–decoder architecture, interconnected with skip connections. Conceptually, the encoder path is used to aggregate semantic information at the cost of reduced spatial information. The decoder path is the counterpart of the encoder that reconstructs the spatial information while being aware of the semantic information learned by the encoder. Skip connections are used to transfer feature maps from the encoder to the decoder to enable precise localization of objects, and improved flow of gradients during backpropagation.

**Figure 2:**
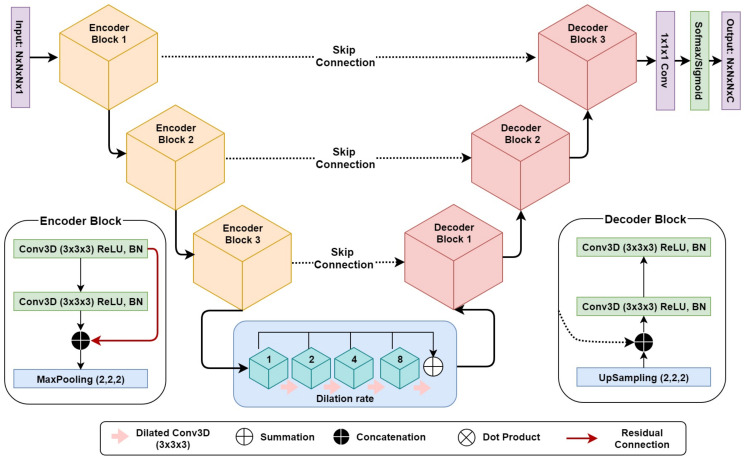
This figure illustrates the architecture of the 3D Dilated Residual U-Net (3D DR-UNet) comprising encoder/decoder blocks. The bottleneck comprises four convolution layers with successively increasing dilation rates from 1–8, to increase the receptive field size. In the encoder block, the red arrow represents the residual connection between convolution layers.

3D DR-UNet (refer to Figure 2) comprises three encoder and decoder blocks, separated by a bottleneck block (Figure 2). This is followed by a 1 × 1 × 1 convolution layer and a sigmoid/softmax-classifier. The architecture includes skip connections between all encoder and decoder blocks at the same spatial resolution. Each encoder/decoder block consists of two 3D convolution layers, where each convolution layer is proceeded by a batch-normalization and a Rectifier Linear Unit (ReLU) layer. In each encoder–convolution block, the input of the first convolution layer concatenated with the output of the second convolution layer (red line in Figure 2). The subsequent 3D max-pooling layer reduces the dimensions of the volume by half. The use of residual connections [29] between convolution layers of each block in the encoder, help improve the flow of gradients in the backward pass of the network. Image dimensions are preserved between the encoder–decoder branches following convolutions, by zero-padding the estimated feature maps. It enabled corresponding feature maps to be concatenated between the branches. We use 1 × 1 × 1 convolution to aggregate the feature maps from the final decoder block. This operation improves discriminative power as feature maps with lower activations are more likely to be suppressed through the assignment of lower weights. Ultimately, a sigmoid activation function was used in the last layer of the first network to produce a value between 0 and 1 for binary segmentation, and a soft-max layer was used for multi-label segmentation, to distinguish the background from the foreground classes. Our network takes a volume $f_{s}$ as input with an image size of N×N×N×1 and produces a segmentation mask of N×N×N. Compared to 3D U-Net, we replace the bottleneck convolution layers of the network with dilated convolutions [30] of size 3×3×3, to enlarge the receptive field and enable the network to capture both local and global contextual information. The dilation rate of the four convolution layers is increased successively from 1–8, and subsequently, their feature maps are summed together, enabling the network to capture the entire volume’s field of view.

Due to GPU memory constraints, it is usually infeasible to process the entire 3D image volume during training. A typical solution is to downsample the original images to a manageable size, however, this adversely impacts the overall segmentation accuracy. Another solution adopted in previous studies is based on a sliding-window, which crops the original images into small blocks and performs segmentation in a block-wise manner. In this study, we propose two different methods for cardiac MRI localization and segmentation, namely, a ‘multi-stage’ and an ‘end-to-end’ network. The former in particular is designed to address this issue by localizing and cropping the ROI, thereby reducing the computational expense.

**Multi-stage localisation and segmentation:** This setup comprises three modules—(a) localization: an encoder–decoder network (based on the 3D DR-UNet) is first used to localize the structure of interest; (b) GCAM: The coarse feature maps produced by the localizer is used by this module to estimate gradient-weighted class activation maps (or GCAM) [31], which is a generalization of class activation (CAM) [32], that provides localized density maps. GCAM discovers the class-specific contribution of each pixel in the feature maps of the final convolution layer to the classification score, based on their corresponding gradients. We compute the gradients of the target class score with respect to the feature maps *x* and then sum the feature maps along the channel axis, weighted by these gradients. The estimation of GCAM for each target class *c* can be expressed as follows:

(1) $$GCAM_{c}=RELU\left(\sum_{k}x_{k}\times \frac{\partial S^{c}}{\partial x_{k}}\right),$$

where $x_{k}\in {I\;R}^{Z,W,H}$ is the *k*^th^ channel of the feature map *x*, and $S^{c}$ is the classification score of class *c*. *Z*, *W* and *H* are the depth, width, and height of image respectively. The estimated GCAM is subsequently used to crop a small ROI, thereby reducing the overall computational expense for the subsequent segmentation step; and (c) segmentation: the cropped ROI is used by the segmentation network (also based on the 3D DR-UNet), to segment the region(s) of interest at a high resolution. While the localization network utilizes the downsampled, low-resolution versions of the original images, the segmentation network can process the cropped ROIs at a higher resolution. This multi-stage pipeline helps mitigate the expensive memory requirements common to the use of 3D convolution kernels, with large medical image volumes.

**End-to-end localisation and segmentation:** the multi-stage approach trains each network, for localisation and segmentation, independently. To evaluate the benefits of such an approach, and in particular, the use of GCAM for localization and ROI-refinement, we also investigated the end-to-end training of both networks, to perform both tasks simultaneously. Given a final feature map $F_{l}\in {I\;R}^{Z,W,H,C}$ estimated by the first network, the input to the second network $F_{s}^{\prime }$, was estimated as: $F_{s}^{\prime }=I_{l}⊗F_{l}$, where, ⊗ denotes element-wise multiplication. This operation helps guide the attention of the second network to regions with high activations, thereby focusing the segmentation on areas suggested by the localisation network.

### 3.1. Loss Functions

We use two different loss functions to train the multi-stage and end-to-end networks.

**BCE-Dice Loss:** The dice coefficient (DC) loss (Equation (2)) is a measure of overlap widely used for training segmentation networks [33]. We used a combination of binary cross-entropy (Equation (3)) and DC loss functions, to train both networks for the binary segmentation task. This combined loss (Equation (4)) is less sensitive to class imbalance in the data, and leverages the advantages of both loss functions. Our experiments demonstrated better segmentation accuracy when using the combined loss for binary segmentation, relative to employing either individually.

(2) $$\zeta_{dc}\left(y,\hat{y}\right)=1-\frac{\sum_{n}y_{nk}{\hat{y}}_{nk}}{\sum_{n}y_{nk}+\sum_{n}{\hat{y}}_{nk}}$$

(3) $$\zeta_{bce}\left(y,\hat{y}\right)=-\sum_{k}\left[{\hat{y}}_{nk}log\left({\hat{y}}_{nk}\right)+\left(1-y_{nk}\right)\left(1-{\hat{y}}_{nk}\right)\right]$$

(4) $$\zeta \left(y,\hat{y}\right)=\zeta_{dc}\left(y,\hat{y}\right)+\zeta_{bce}\left(y,\hat{y}\right)$$

In Equations (2) and (3) ${\hat{y}}_{nk}$ denotes the output of the model, where *n* represents the pixels, *k* denotes the classes, and the ground truth labels are denoted $y_{nk}$. We used the two-class version of the DC loss $\zeta_{dc}\left(y,\hat{y}\right)$ proposed in [30].

**Multi-class Dice loss:** A modified version of the soft-dice loss was used for the task of multi-class segmentation. We first calculate the dice score for each class individually, and then average them over the number of classes. In order to segment an N×N×N input image (for example, a Cine-MRI volume with LV, RV, myocardium (MYO) and Background as labels), the outputs are four probabilities for classes k=0,1,2,3 where, $\sum_{c}y_{n,k}=1$ for each voxel. Given the one-hot encoded ground truth label ${\hat{y}}_{n,k}$ for that corresponding voxel, the multi-class soft dice loss is defined as follows:

(5) $$\zeta_{dc}\left(y,\hat{y}\right)=1-\frac{1}{K}\left(\sum_{k}\frac{\sum_{n}y_{nk}{\hat{y}}_{nk}}{\sum_{n}y_{nk}+\sum_{n}{\hat{y}}_{nk}}\right).$$

### 3.2. Datasets

**Figure 3:**
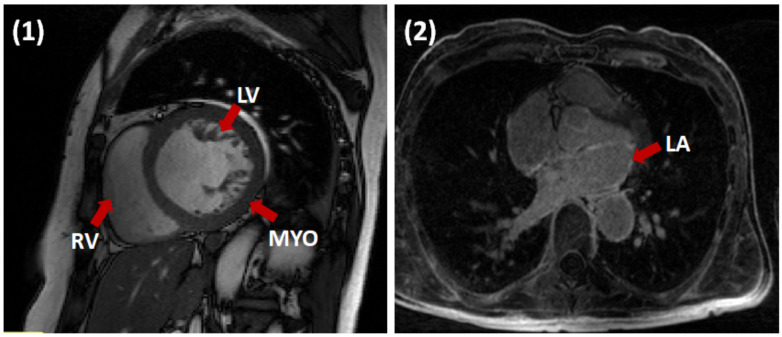
Examples of Cine short-axis (left) and LGE-MR (right) images with different heart structures. The red arrow points toward specific heart structure in different moralities.

In order to validate our framework, we conducted experiments using two different datasets comprising Late-Gadolinium Enhanced MRI (LGE-MRI) and Cine-MRI scans. Both datasets are publicly available and were provided as part of the STACOM 2018 and 2017 challenges. This study complies with the Declaration of Helsinki. For all patients, informed consent obtained and the institutional ethical board (ethics committee of the University of Utah and University Hospital of Dijon) approval was provided prior to the study. The data are parts of the challenge and analyzed retrospectively. Table 1. demonstrates imaging parameters of the two datasets employed in this study as well as pathology. Figure 3 illustrates a sample slice from each dataset and the specific heart structure respectively. We split both the datasets into training, validation (80%), and test (20%) sets. On the 80% training data, we performed five-fold cross-validation, and then tested the models on the remaining 20%. We tried to be fair and have a thorough evaluation of our model, and that is why we split the data in this way. Since the challenge organizers only provided the ground-truth label for their training sets but not test set.

#### 3.2.1. LASC STACOM 2018

This dataset comprises 100 3D LGE-MRIs acquired from patients diagnosed with atrial fibrillation (AF), and was provided as part of the STACOM 2018 challenge for the task of left atrium (LA) segmentation. The resolution of the provided data is 0.625×0.625×0.625 mm^3^, with dimensions of 88×640×640 and 88×576×576 voxels. A large proportion of the data was provided by the University of Utah [34], while the rest were from multiple other institutes. Each 3D LGE-MRI volume was acquired using a clinical whole-body MRI scanner (either a 1.5 Tesla Avanto or 3.0 Tesla Verio), and its corresponding ground truth binary mask for the LA cavity, was annotated by experts.

#### 3.2.2. ACDC STACOM 2017

The ACDC dataset provided as part of the MICCAI 2017 Challenge on automated cardiac diagnosis, was created from real clinical exams, acquired at the University Hospital of Dijon. This dataset consists of cardiac Cine-MR images (CMRI) from 100 patients that belong to one of five classes, namely, healthy, dilated cardiomyopathy, hypertrophic cardiomyopathy, heart failure with infarction, and right ventricular abnormality. The provided images are uniformly distributed over all classes. Ground truth segmentations for the LV cavity, RV endocardium, and MYO, at end-diastole (ED) and end-systole (ES), were generated manually by experts for all 100 samples. For each patient, short-axis (SA) CMRIs with 28–40 frames are available, in which the ED and ES frame have been annotated. On average images, consist of nine slices where each slice has a spatial resolution of ≈235 × 263 voxels. The image slices cover the LV from the base to the apex. In-plane voxel spacing varies from 1.37 to 1.68 mm, with a slice thickness of 5–10 mm. These images were acquired over six years using two MRI scanners of different magnetic strengths (1.5 T (Siemens Area, Siemens Medical Solutions, Germany) and 3.0 T (Siemens Trio Tim, Siemens Medical Solutions, Germany)). Cine-MR images acquired during a breath-hold, with retrospective or prospective gating along the short-axis.

### 3.3. Training

Due to low contrast in some of the LGE-MRI and Cine-MRI volumes, we enhanced the contrast slice-by-slice, using contrast limited adaptive histogram equalization (CLAHE) [35], and normalized each volume based on its the mean and standard deviation of intensity values. The volumes were zero-padded, and center-cropped to a fixed-size input for the networks. Both datasets were split into 80% for training and validation and 20% for testing within a five-fold cross-validation scheme. For the ACDC dataset, we uniformly distributed the pathological cases across the training and validation sets. In all experiments, the trained networks evaluated using the held-out test data (20 subjects). The adaptive moment estimation (ADAM) optimizer [36], a type of gradient descent algorithm, was used to estimate the weights of all networks throughout this study. The learning rate was fixed at 0.0001, and the exponential decay rates of the 1st and second—-moment estimates were set to 0.9 and 0.999, respectively. During training, segmentation accuracy was evaluated on the validation set after each epoch of the network. Networks trained until the validation accuracy stopped increasing, and the best performing model was selected for evaluation on the test set. The network was developed in Keras and TensorFlow [37], an open-source deep learning library for Python, and trained on an NVIDIA Titan X-Pascal GPU with 3840 CUDA cores and 12GB RAM.

### 3.4. Evaluation Metrics

To evaluate the performance of each CNN, we compared their segmentations with the provided ground-truth masks, for each MRI volume. We used three different metrics to evaluate segmentation accuracy, namely, the dice similarity coefficient (DSC), Hausdorff distance (HD), and average surface distance (ASD). The DSC metric, also known as the F1-score, measures the degree of overlap between the predicted and ground truth segmentations. It is the most widely used metric for evaluating segmentation quality in medical imaging [38], and is defined as:

(6) $$DSC\left(G,P\right)=\frac{2TP}{\left(FP+2TP+FN\right)}=\frac{2|G_{i}\cap P_{i}|}{|G_{i}\left|+\right|P_{i}|},$$

where *G* is the ground truth, and *P* is the predicted mask. TP, TN, FP and FN refer to true positive, true negative, false positive, and false negative voxels, respectively.

HD is defined as the maximum of the minimum voxel-wise distances between the ground truth and predicted object boundaries. This is expressed as:

(7) $$HD\left(G,P\right)=\max_{g\in G}\left\{\min_{p\in P}\left\{\sqrt{g^{2}-p^{2}}\right\}\right\}.$$

ASD is the average of the minimum voxel-wise distances between the ground truth and predicted object boundaries. By defining the shortest Euclidean distance of an arbitrary voxel *v* to a point *P* as $\bar{d}\left(v,P\right)=\min_{p\in P}||v-{p||}_{2}$, ASD can be expressed as:

(8) $$ASD\left(G,P\right)=\frac{1}{N_{G}+N_{P}}\left\{\sum_{x_{p}\in P}\bar{d}\left(x_{P},G\right)+\bar{d}\left(x_{G},P\right)\right\},$$

where $N_{P}$ and $N_{G}$ are the number of voxels on the object boundaries in the predicted and ground truth masks, respectively.

## 4. Results

The LASC dataset comprised 100 volumes of 3D Late-Gadolinium Enhanced MRIs, from patients diagnosed with atrial fibrillation. The CNN-based segmentation algorithms investigated in this study were trained using a subset of this data, to learn features relevant to localizing and segmenting the left atrium. All networks trained across these cross-validation experiments were evaluated using the held-out test data (20 samples). The segmentation performance of each network investigated was assessed using three evaluation metrics, namely, dice score (DSC), Hausdorff distance (HD), and average surface distance (ASD). These measures were averaged across all cross-validation experiments, and are summarized in Table 2.

The 3D DR-UNet multi-stage approach achieved mean DSC, HD, and ASD values of 90.44%, 21.68 mm, and 1.53 mm on the test data, respectively. On the other hand, the 3D DR-UNet end-to-end network achieved a mean DSC value of 88.92%, HD of 21.73 mm, and ASD of 1.62 mm. The segmentation scores for the other three networks were as follows—3D U-Net: DSC of 84.33%, HD of 25.56 mm, ASD of 1.83 mm; 3D-VNet: DSC of 84.26%, HD of 26.39 mm, ASD of 1.62 mm; and 3D DR-UNet: DSC of 87.46%, HD of 24.61 mm, ASD of 1.72 mm. These results indicate that both proposed approaches, i.e., the multi-stage and end-to-end networks, outperform the state-of-the-art in terms of the DSC, HD, and ASD metrics. Furthermore, based on these results, we conducted additional experiments using the 3D DR-UNet multi-stage approach, with higher network capacity in the second stage (i.e., 16 filters rather than 8). These results also presented in Table 2 and highlight the additional improvement in segmentation accuracy afforded by increasing network capacity. The DSC, HD, and ASD metrics improved to 91.2%, 20.3 mm, and 1.38 mm, respectively.

For the ACDC dataset, we resampled all volumes to 10 × 1.25 × 1.25 mm per voxel to account for varying spatial resolutions. The intensities of all images normalized to zero-mean and unit variance. Subsequently, we zero-padded all volumes to 16 × 256 × 256 voxels and used them to train all networks. Table 3 summarizes the average segmentation performance of different methods across all structures. As with the previous dataset, the DSC, HD, and ASD metrics were used to evaluate segmentation quality. In the first set of experiments on this dataset, all networks performed the max pooling operation on all three dimensions of the input volumes. The 3D DR-UNet multi-stage achieved a mean DSC score of 84.8%, HD of 5.81 m, and ASD of 0.57 mm, on the test data. While, the end-to-end variant achieved mean DSC scores of 83.7%, HD of 5.99 mm, and ASD of 0.60 mm. As the slice thickness along the *z* dimension of all volumes in the ACDC dataset is very large, 5–10 mm, in the second set of experiments, we ensured that the inputs were downsampled only along the *x* and *y* axes. With these settings, 3D DR-UNet multi-stage yielded a mean DSC score of 88.4%, HD of 4.91 mm and ASD of 0.41 mm, and the end-to-end variant achieved mean DSC scores of 87.5%, HD of 5.58 mm, and ASD of 0.54 mm, respectively. These results indicate that both proposed methods once again outperformed the state-of-the-art, and both benefit from max-pooling only along the *x* and *y* dimensions, to account for the large voxel sizes along the *z* axis. Additionally, structure-wise segmentation accuracy for the RV, LV, and MYO in the ACDC dataset, is summarized in Table 4. These results further highlight the improvement in segmentation quality afforded by 3D DR-UNet multi-stage (Keep Z), relative to all other networks investigated. The former consistently outperforms the rest, across all structures, at both the ED and ES phases.

**Figure 4:**
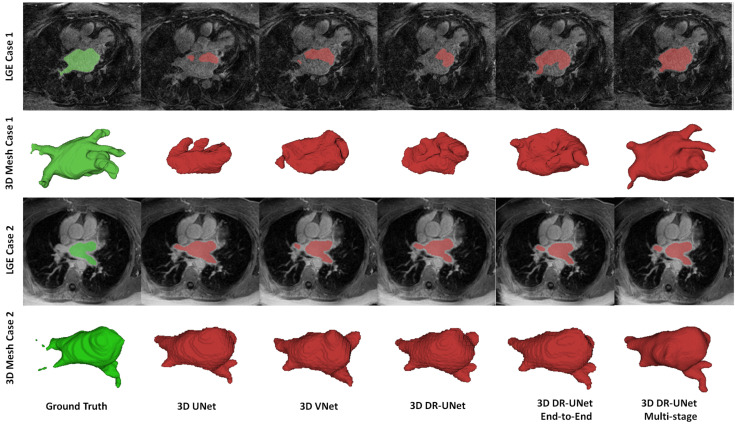
Examples of LA segmentations and 3D mesh generated using each network for the LASC dataset. Top and bottom rows depict slices chosen at random from two test cases and their mesh representation. The ground-truth mask is in green, and the rest are in red color respectively.

**Figure 5:**
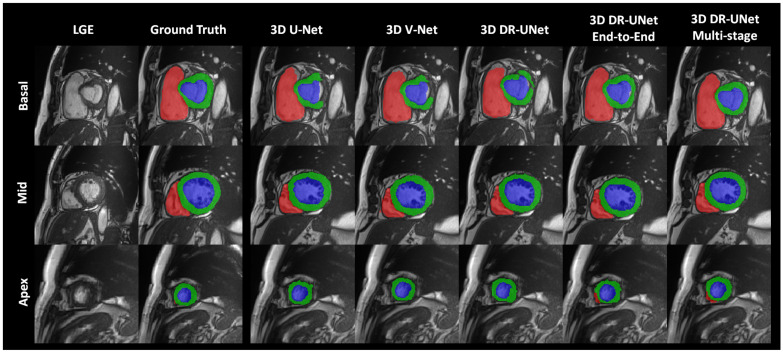
Figure depicts segmentation of the RV, LV, and MYO at the ED phase, for a test sample from the ACDC dataset, generated using each network. Rows from top to bottom depict the basal, middle and apical slices, respectively. In all figures, red, blue and green represent the RV, LV, and MYO, respectively.

**Figure 6:**
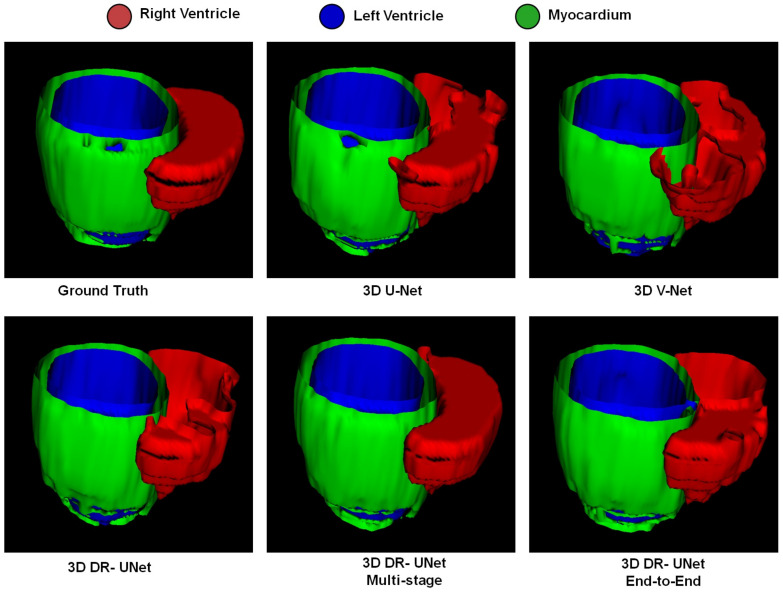
The surface mesh outputs produced by the different networks on a random test subject at the ED phase of the heart. The colors red, green and blue indicate RV, MYO, and LV respectively.

The quality of the segmentations generated using each network investigated in this study can be visually assessed in Figure 4 for the LASC dataset, and Figure 5 and Figure 6 for the ACDC dataset. These help further highlight the benefits of the proposed multi-stage approach, for example, Figure 6 clearly shows the reduced number of false positives and false negatives estimated using the former, compared to all other network configurations. This leads to RV, LV and MYO surfaces that resemble the ground truth more closely than the rest; Figure 4 highlights a similar advantage for the LA; and Figure 5 depicts a test sample wherein, the myocardium is captured completely in the apical and basal slices using the multi-stage approach, which is not afforded by the others. This is particularly important in the analysis of cardiac MRI as accurate segmentation of these slices directly impacts the estimation of important cardiac functional indices such as LV volume, used routinely in the clinic for the diagnosis of various cardiac pathologies. Furthermore, Figure 4 illustrates that the proposed methods accurately segment the LA in contrast to the others, particularly in the case of the first subject which was more challenging due to the presence of noise and low soft tissue contrast.

**Comparison with State-of-the-art Methods:** We further compared our proposed method with two other similar localization and segmentation methods including, Yang et al. and Mask-RCNN. The results are shown in Table 2 and Table 3. In [22], the authors first trained a Faster R-CNN to find the ROIs (left atrium) within the volume and further segments LA using an encoder–decoder. They employed many tweaks on their segmentation backbone such as, deep supervision, combo loss, and using transfer learning to improve their model initialization. They also adopted a recursive refinement step to improve the segmentation accuracy further. Thereby, their pipeline has so many trainable parameters, and it is very complex to train. In contrast, we aimed to introduce a light-weight network architecture to perform both localization and segmentation together. Here, our localization network has less than a million parameters, which are very fast to train, and inference time takes only less than a second for the entire volume. Yang et al. approach without any other tweaking steps achieved an overall dice score of 0.893 and HD and ASD values of 24.45 and 1.455 mm for 20 test subjects on the LASC dataset, while our method achieved an overall dice score of 0.91. Our reported results are an average of all five fold-cross-validation, which covers both hard and easy cases. We also compared our model with Mask-RCNN for the ACDC dataset, which outperformed Mask-RCNN based on all evaluation metrics. The Mask-RCNN method achieved an overall dice score of 0.853 and HD and ASD values of 5.127 and 5.05 mm, respectively. These results highlight the advantages afforded by our proposed method.

### Statistical Analysis

The effectiveness of our approach and its advantages over other segmentation methods was evaluated using Bland–Altman analysis [41]. This statistical technique determines the agreement between two quantitative measurements by constructing limits of agreement (LoA). The limits are estimated using the mean and the standard deviation of the differences between the two segmentation methods. For each component in the plot (each dot corresponds to one MR scan), a negative bias indicates that the automated segmentation overestimates the volumes, while a positive one indicates that it underestimates the volumes.

**Figure 7:**
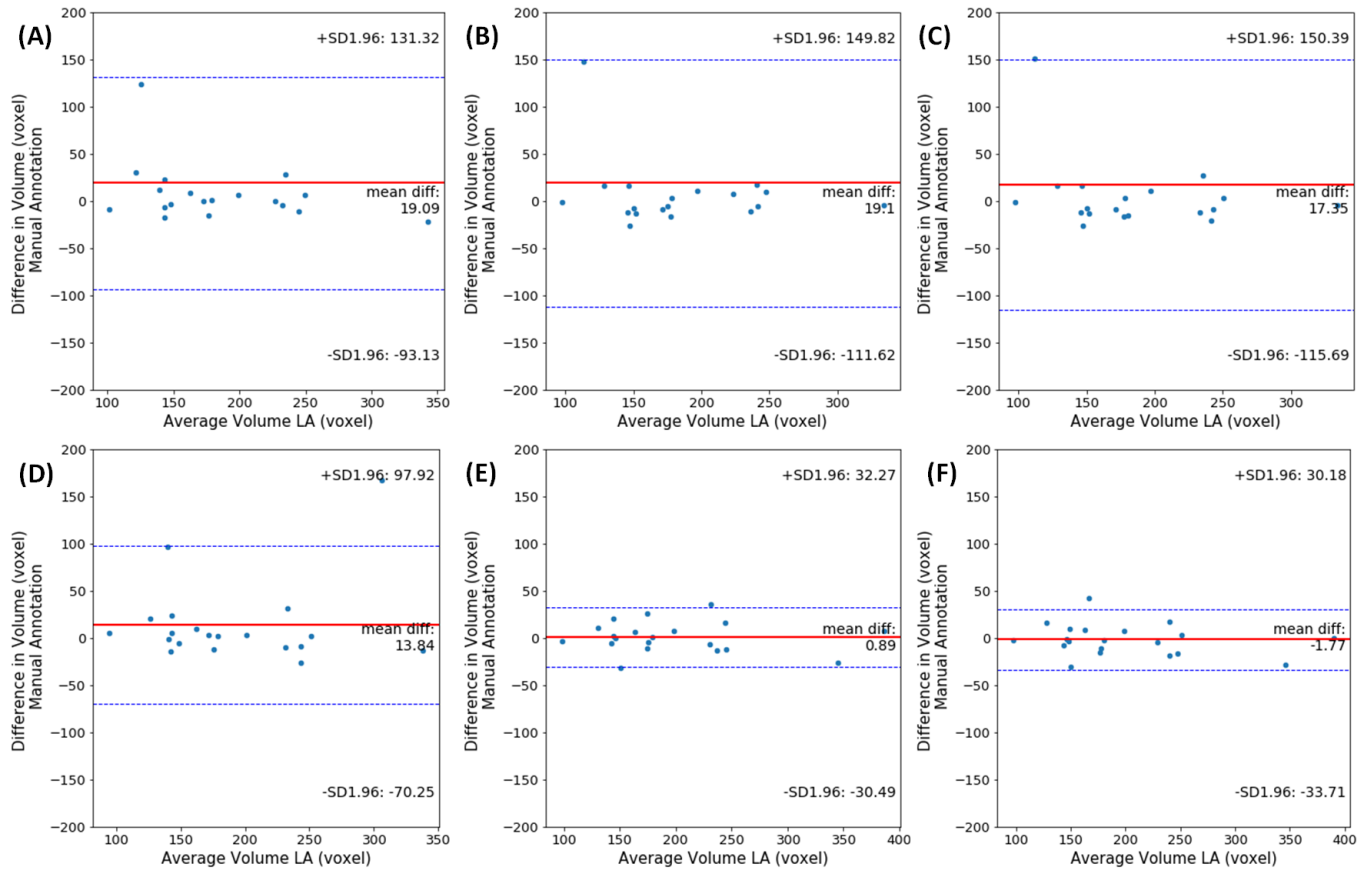
Bland–Altman graphics presenting the differences between volumes obtained by manual and automated segmentation methods, plotted against the mean of the 2 measurements for LASC dataset. Volumes per patient are expressed in mm^3^. The solid lines(red) indicate the mean bias and the dashed lines(blue) show the 95% CI (±1.96 SD). Each plot displays the agreement between method segmentation output against the ground truth, (**A**) 3D UNet, (**B**) 3D VNET, (**C**) 3D DR-UNet, (**D**) 3D DR-UNet End-2-End, (**E**) 3D DR-UNet multi-stage with eight filters and (**F**) 3D DR-UNet multi-stage with 16 filters.

The Bland–Altman plots for differences in LA volumes obtained using manual and automated segmentation methods on the LASC dataset, are shown in Figure 7. The volumes per patient is expressed in mm^3^. It can be observed that the agreement between our proposed method 3D DR-UNet multi-stage (Keep Z) and manually generated ground truth is high with a bias (mean signed difference) of −1.77 mm^3^ and limits of agreement of ±30.18 mm^3^ in terms of LA volume estimation. These results suggest that the proposed method has a small bias to overestimate LA volume and that the variation between automated and manual estimates of LA area is only slightly greater than the expert manual annotation. The other methods have higher average volume differences, and there are some outlier cases (refer to plots A-C in Figure 4), regarded as hard-examples to segment due to the presence of low contrast and noise in the scans. However, our methods produced more accurate segmentation masks for such cases resulting in significantly lower mean differences in LA volume and their corresponding LoA. In other methods, since the global context information is not considered, they tend to over-segment parts of MR volume, which have either similar morphology or contrast to LV, RV, and Myo structures. However, our proposed methods accurately segment those cases, which is mainly because of the localization step in both multi-stage and end-to-end networks that reduces the search space. For the end-to-end approach, the first network produces the shallow probability map of the heart anatomy, and it’s concatenated as an extra channel with the input of the second network to guide the model to focus only on the localized region. It prevents the model from over-segmentation, consequently resulting in a lower number of outlier cases.

**Figure 8:**
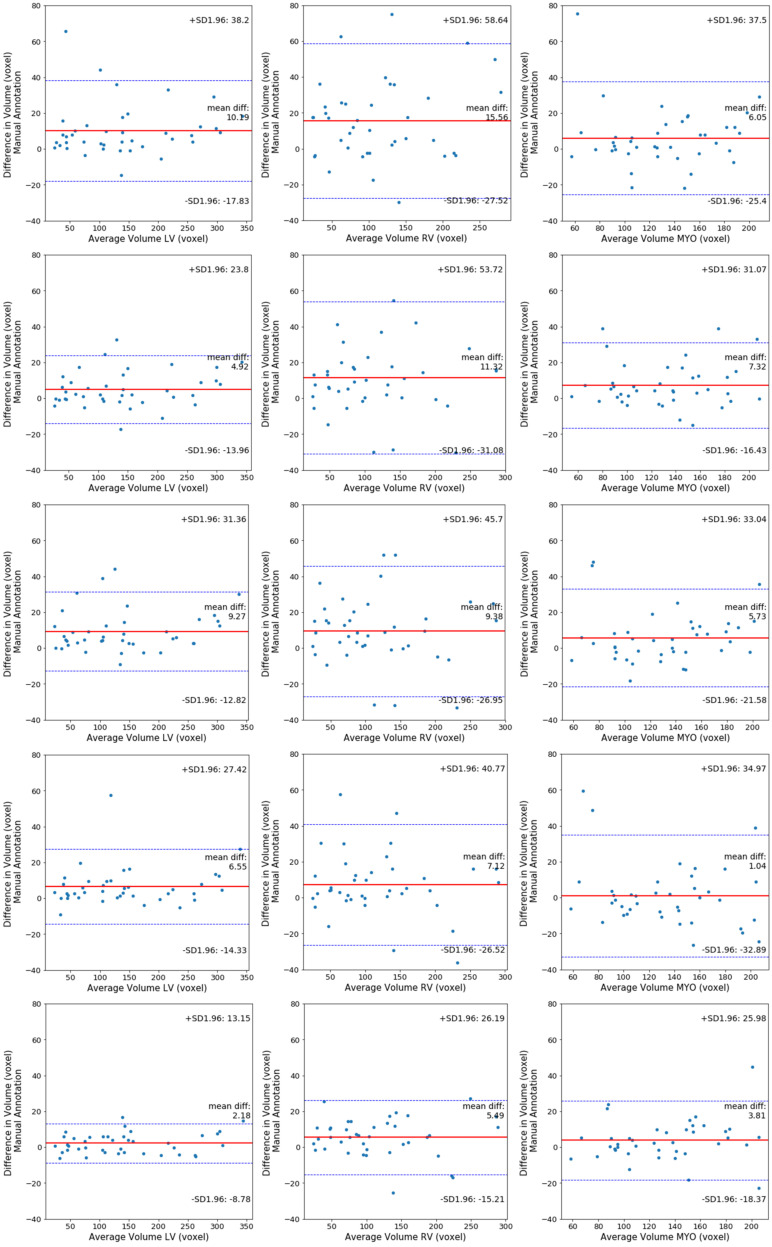
Bland–Altman graphics showing the differences between volumes obtained by manual and automated segmentation plotted against the mean of the 2 measurements for ACDC dataset. Volumes per patient are expressed in mm^3^. The solid lines(red) indicate the mean bias and the dashed lines (blue) show the 95% CI (mean bias 1.96 SD). Each row shows three plots of LV, RV, and Myo for 3D UNet, 3D VNet, 3D DR-UNet, 3D DR- UNet multi-stage (Keep Z) and 3D DR- UNet end-to-end (Keep Z) respectively.

Furthermore, in Figure 8 each row depicts three Bland–Altman plots of the LV, RV, and Myo respectively, generated with respect to the ground truth, for each segmentation method investigated. These plots indicate that the multi-stage and end-to-end methods have a higher agreement with the ground truth across all three cardiac structures, compared with 3D UNet, 3D VNet, and 3D DR-UNet. 3D DR- UNet multi-stage (Keep Z) has an average mean difference volume of 2.18 mm^3^ for LV, 5.49 mm^3^ for RV, and 2.81 mm^3^ for MYO, with respect to their respective ground truth segmentations.

## 5. Discussion

Automatic localization and segmentation of cardiac structures in MRI are essential for several clinical applications as they heavily influence subsequent quantitative and diagnostic analyses. Although fully automated systems for extracting cardiac functional indices from MRIs are yet to be adopted into routine clinical examinations, the design of robust frameworks for the same has the potential to enhance the overall diagnostic workflow. For example, cardiac MRI segmentation is necessary to measure blood pool and cardiac volumes, which aid in deriving functional indices such as ES and ED ventricular volumes, ejection fractions, etc. These quantitative measures in turn are used to diagnose patients. Consequently, automating this process would substantially reduce the time spent by cardiologists in analyzing one patient’s images, as currently, the heart is segmented either manually or semi-automatically to compute these measures. Additionally, this would help reduce inter- and intra-rater variations, which are inevitable when such analyses are done manually, thereby streamlining the overall process. Furthermore, intervention planning and subsequent image-guided interventions could benefit significantly from the availability of high-quality volumetric segmentation. To address these needs, this paper proposes a multi-stage, and end-to-end fully convolutional network for cardiac MRI segmentation, which enables automated and robust delineation of cardiac structures in the presence of varying degrees of pathology-induced morphological changes to the heart.

The flexibility of the framework is demonstrated by evaluating its segmentation performance on two different datasets, acquired at separate centers, with different scanners and protocols. The proposed framework, and in particular the multi-stage variant, consistently outperformed the state-of-the-art in terms of segmentation accuracy. While CNN-based segmentation algorithms have proven to be highly effective for a variety of applications, they are computationally expensive and require a large amount of annotated training data to generalize well to unseen samples. It is particularly challenging to address within the medical domain, due to the high cost of acquiring such ground truth annotations. Additionally, 3D medical images such as cardiac MRI volumes require a lot of computational resources (memory) due to their large size, limiting the complexity of trainable networks. Consequently, we designed our multi-stage network to exploit the benefits of localizing the structure(s) of interest (in its first stage), using gradient-based class activation maps (GCAM). By identifying ROIs, sub-volumes within the cardiac MRIs were cropped and used to train the segmentation network. This in turn reduced the computational expense, thereby enabling the design of a deeper segmentation network while also maintaining the sub-volumes at a higher resolution, relative to when the entire image volume is used. It helps reduce the loss in information resulting from downsampling the image volumes. The proposed multi-stage approach also exploits both local and global information using dilated convolutions and residual connections in the encoder branch. Dilated convolutions control the spatial context of the features learned by the model and adjust the filter’s receptive-field to capture multi-scale information. Furthermore, the residual connections between convolution layers ensure a smoother flow of gradients in the backward pass.

Experiments conducted using the LASC dataset indicate that end-to-end training of our framework produces comparable results to its multi-stage variant. It is because the attention of the second encoder–decoder network is guided by the features learned in the first network. Both approaches exploit the benefit of intermediate localization of the LA, resulting in improved segmentation accuracy, relative to the other approaches investigated, which lack this component. For the ACDC dataset, the multi-stage method substantially outperformed the end-to-end network across all three structures. It is because the LA is relatively easy to segment in the provided LGE-MRI dataset due to the high resolution of the images. Consequently, the advantage offered by the multi-stage approach (i.e., segmenting at a higher resolution) is less apparent than in the ACDC dataset (where the spatial resolution of the images is lower). We believe that the proposed framework is useful for a variety of clinical applications requiring cardiac MRI segmentation, by providing high-quality segmentation for images acquired from a wide age demographic of patients, with varying degrees of pathology-induced morphological changes, and at different centers, using different scanners and protocols.

Even though the segmentation accuracy of our approach was high for the LV blood pool, RV and LA, there is still room for improvement, especially for the myocardium and LA borders. For future work, we aim to test and verify the robustness and generalizability of our method by evaluating the performance on a more diverse and motion included clinical MR images. The challenge datasets are typically collected carefully to have better results reproducibility. However, in the real clinical scenario, clinicians acquire many low and high-quality cardiac MR images under different settings and configurations. On the other hand, deep learning-based models are sensitive to domain shift and data acquisition change. Therefore, a comprehensive evaluation of the proposed model will be performed using the UK BioBank dataset, which includes a large number of subjects, with different pathologies and artifacts.

## 6. Conclusions

In this paper, we introduced two strategies for training a segmentation network, namely, multi-stage and end-to-end, for automatic cardiac MRI localization and segmentation. Accurate heart chamber localization directly enables faster data navigation and visualization of target structures, which can undoubtedly save the radiologist times. The objective was to facilitate the generation of accurate, high-resolution segmentation with limited training samples. Our approach does not require significant pre-processing of the MR images or post-processing operations and utilizes the entire 3D volume, to explicitly use spatial information in three dimensions. The proposed method first estimates a coarse density map localizing the structure of interest. It acts as an attention map focusing the second (segmentation) stage of the network within the region of interest (ROI). Finding ROIs with high precision allows us to build a deeper network, and also segment cardiac structures at a higher resolution. Compared to existing CNN-based segmentation approaches, the proposed scheme has shown its superiority in terms of segmentation robustness and accuracy, for images exhibiting substantial variations in morphology and appearance due to pathology.

## Figures and Tables

**Table 1:** Imaging parameters of the sequences employed in this study. Abbreviations: FOV, field of view.

Datasets	Sequence	Resolution (mm^3^)	Slices	FOV (mm^3^)	MR Scanner	Pathology
ACDC STACOM	Short-axis MR	5–10 × 1.37 × 1.68	9–18	9–18 × 235 × 263	1.5 T Area 3.0 T Trio	Healthy, Cardiomyopathy, Hypertrophies, Infraction
LASC STACOM	LGE-MR	0.625 × 0.625 × 0.625	88	88 × 640 × 640 88 × 576 × 576	1.5 T Avanto 3.0 T Verio	Atrial Fibrillation

**Table 2:** Segmentation performance of different network architectures on the test samples from the LASC STACOM 2018 dataset, evaluated using the dice similarity coefficient (DSC), Hausdorff distance (HD) and average surface distance (ASD) metrics (mean ± (std)). Filters denote here the initial number of convolution kernels which used the byeach network.

Methods	DSC ↑	HD [mm] ↓	ASD [mm] ↓
	**8 Filters**		
3D U-Net [17]	0.843 (±0.187)	25.569 (±21.107)	1.853 ±(1.536)
3D V-Net [33]	0.842 (±0.209)	26.397 (±25.387)	1.628 (±1.136)
3D DR-UNet [39]	0.874 (±0.098)	24.612 (±11.829)	1.728 (±1.200)
3D DR-UNet multi-stage	0.904 (±0.029)	21.685 (±7.793)	1.539 (±0.615)
3D DR-UNet end-to-end	0.889 (±0.062)	21.734 (±11.216)	1.621 (±0.889)
	**16 Filters**		
Yang et al. [22]	0.893 (±0.067)	24.45 (±12.455)	1.455 (±1.211)
3D DR-UNet multi-stage	**0.912 (±0.031)**	**20.308 (±8.305)**	**1.386 (±0.486)**

**Table 3:** Segmentation performance of different network architectures on the ACDC STACOM 2017, evaluated using the DSC, HD and ASD metrics (mean ± (std)). Filters denote the initial number of convolution kernels used by each network.

Methods	DSC ↑	HD [mm] ↓	ASD [mm] ↓
	**32 Filters**		
3D U-Net [17]	0.825 (±0.123)	7.282 (±7.013)	1.680 (±4.505)
3D V-Net [33]	0.842 (±0.080)	5.894 (±2.664)	0.586 (±0.406)
3D DR-UNet [39]	0.848 (±0.056)	5.729 (±2.573)	0.591 (±0.461)
Mask-RCNN [40]	0.853 (±0.088)	5.127 (±2.312)	0.505 (±0.821)
3D DR-UNet multi-stage	0.850 (±0.068)	5.815 (±2.715)	0.575 (±0.465)
3D DR- UNet end-to-end	0.837 (±0.078)	5.991 (±2.598)	0.608 (±0.370)
3D DR-UNet multi-stage (Keep Z)	**0.884 (±0.053)**	**4.916 (±2.247)**	**0.415 (±0.244)**
3D DR-UNet end-to-end (Keep Z)	0.875 (±0.084)	5.583 (±3.447)	0.543 (±0.651)

**Table 4:** Structure-wise DSC, HD and ASD scores (mean ± (std)) for the right ventricle (RV), left ventricle (LV) and myocardium (MYO) from the ACDC dataset.

Methods	LV			RV			MYO		
	DSC ↑	HD [mm]↓	ASD [mm] ↓	DSC↑	HD [mm]↓	ASD [mm]↓	DSC↑	HD [mm]↓	ASD [mm]↓
	**32 Filters**								
3D U-Net [17]	0.889 (±0.128)	3.943 (±2.69)	0.568 (±0.86)	0.781 (±0.203)	11.751 (±19.97)	3.780 (±16.82)	0.805 (±0.113)	6.153 (±3.55)	0.693 (±1.08)
3D V-Net [33]	0.908 (±0.066)	3.683 (±1.522)	0.424 (±0.25)	0.809 (±0.121)	8.231 (±4.25)	0.777 (±0.70)	0.809 (±0.079)	5.769 (±2.43)	0.556 (±0.31)
3D DR-UNet [39]	0.904 (±0.081)	3.588 (±1.54)	0.439 (±0.32)	0.825 (±0.109)	8.097 (±3.78)	0.78 (±0.69)	0.814 (±0.098)	5.501 (±2.64)	0.550 (±0.42)
3D DR- UNet multi-stage	0.910 (0.090)	3.634 (±2.39)	0.422 (±0.47)	0.820 (±0.107)	8.046 (±5.79)	0.789 (±1.21)	0.821 (±0.131)	5.766 (±3.19)	0.513 (±0.62)
3D DR- UNet end-to-end	0.901 (0.080)	3.799 (±2.47)	0.474 (±0.46)	0.811 (±0.120)	8.166 (±6.11)	0.776 (±1.23)	0.800 (±0.201)	6.007 (±4.19)	0.575 (±0.71)
3D DR- UNet multi-stage (Keep Z)	**0.928** **(±0.057)**	**3.054** **(±1.24)**	**0.313** **(±0.17)**	**0.871** **(±0.075)**	**6.975** **(±3.44)**	**0.515** **(±0.41)**	**0.853** **(±0.06)**	**4.718** **(±2.32)**	**0.418** **(±0.19)**
3D DR- UNet end-to-end (Keep Z)	0.920 (0.070)	3.623 (±2.39)	0.397 (±0.41)	0.86 (±0.097)	7.889 (±5.59)	0.727 (±1.11)	0.846 (±0.108)	5.237 (±3.09)	0.504 (±0.60)

## Abbreviations

The following abbreviations are used in this manuscript:

CMR	Cardiac magnetic resonance
CVDs	Cardiovascular diseases
LV	Left ventrical
RV	Right ventrical
Myo	Myocardium
ACDC	Automatic cardiac segmentation challenge
LASC	Left atrium segmentation challenge
3D DR-UNet	3D dilated residual U-Net
GCAM	Gradient-weighted class activation maps
